# Surgical Outreach for Children by International Humanitarian Organizations: A Review

**DOI:** 10.3390/children4070053

**Published:** 2017-06-28

**Authors:** J. Matthew Kynes, Laura Zeigler, Kelly McQueen

**Affiliations:** 1 Department of Anesthesiology, Monroe Carell, Jr Children’s Hospital at Vanderbilt, Nashville, TN 37209, USA; laura.zeigler@vanderbilt.edu; 2 Department of Anesthesiology, Vanderbilt University Medical Center, Nashville, TN 37209, USA; kelly.mcqueen@vanderbilt.edu

**Keywords:** global health, pediatric surgery, humanitarian outreach, missions

## Abstract

Low- and middle-income countries carry a disproportionate share of the global burden of pediatric surgical disease and have limited local healthcare infrastructure and human resources to address this burden. Humanitarian efforts that have improved or provided access to necessary basic or emergency surgery for children in these settings have included humanitarian assistance and disaster relief, short-term surgical missions, and long-term projects such as building pediatric specialty hospitals and provider networks. Each of these efforts may also include educational initiatives designed to increase local capacity. This article will provide an overview of pediatric humanitarian surgical outreach including reference to available evidence-based analyses of these platforms and make recommendations for surgical outreach initiatives for children.

## 1. Introduction

Medical and technological advances over the past 100 years have produced vast gains in the prospects for the health and well-being of children worldwide. Improvements in sanitation, widespread vaccination, improved food supplies, control of infectious diseases, improved road safety and other interventions have extended life expectancy by over 30 years in many developed countries [1]. Since 1990, global under-five mortality has decreased 53% with an acceleration in the annual rate of reduction during that time [1]. These improvements, however, have been unequally distributed. In 2015, child mortality remained 12 times higher in sub-Saharan Africa than in the average high-income country [2]. Children in many low and middle-income countries (LMIC) still face threats to health and development that high-income countries faced in the previous century, particularly in the area of surgical disease.

In LMICs, external, humanitarian efforts often support fragile healthcare infrastructures. Healthcare systems in LMICs have public and private components, and the public components are generally resource constricted, focusing on the largest burden of disease. In LMICs, the majority of social resources are focused in urban centers and this draws a high concentration of care providers and the most sophisticated resources into centralized urban areas, resulting in sparse resources and much fewer trained providers in rural areas. External supports are often directed toward bolstering public facilities and often focus on short-term interventions or improvements. External aid takes many forms, some of which is provided by formal arrangements between multinational organizations or governments and ministries of health, the scale of which is illustrative of the need for external involvement. In low-income countries, external funds account for an average of 28% of national health spending [1]. In 2015, US$6.5 billion in international aid was allocated to support child health, which amounts to 18% of $36.1 billion in total global health funding [3]. The direct financial partnerships between health ministries and large donors are distinct from but may be augmented informally by humanitarian outreach, which is non-reimbursable short- or long-term health assistance provided by international organizations (International Committee of the Red Cross (ICRC); Médecins Sans Frontiéres (MSF)) or smaller charitable, non-governmental organizations (NGOs), and these are the focus of this review. Humanitarian outreach from international organizations or NGOs often focuses on acute needs, frequently following a humanitarian crisis or disaster, but short-term surgical care is also frequently provided by organizations whose mandate is the provision of specific surgical care, and may or may not be geographically recurring.

Recent global health advocacy has focused on surgical systems strengthening as a component of universal health care. This has been brought to the forefront with the publication of Global Surgery 2030, the initial findings of the Lancet Commission on Global Surgery, which reported that 28–32% of the global burden of disease can be attributed to surgically treatable conditions and that 5 billion people lack timely access to safe surgical care [4]. This publication has mobilized a more cohesive approach to expanding surgical services to areas of need in a sustainable fashion and set targets for improving the areas of access, workforce density, surgical volume, safety, and affordability of surgical services worldwide by 2030. Increasingly, surgery is being championed as “an indivisible, indispensable part of health care” [5]. Similarly, the Disease Control Priorities for Developing Countries, 3rd Edition, (DCP3) a publication of the World Bank, included a volume of Essential Surgery [6]. This publication reveals that 44 basic surgical interventions and the related anesthesia are cost-effective for all LMICs, at the level of the First Referral Hospital. This change will dramatically impact access to basic pediatric surgery if the recommendations are implemented. The DCP3 also reviews external surgical platforms and the related costs of short-term surgical missions versus long-term infrastructure building for surgical care [6].

LMICs carry not only a disproportionate burden of unmet surgical disease but also have a greater need for pediatric surgical services. Because of improvements in overall health and longevity in high income countries, the population aged 0–14 years has decreased from 29% to 17% since 1960, while in low-income countries it has remained stable at around 43% during the same period [7]. Projections into the middle of this century show that in the near future, one of every three children younger than 18 years old will live in sub-Saharan Africa [8]. The Millennium Development Goals (MDG) included a target to decrease under-five mortality by two-thirds (MDG 4) which has transitioned into the Sustainable Development Goal (SDG) of ending preventable deaths of children under age 5 years and reducing under-five mortality to below 25 deaths per 1000 live births (SDG 3.2) [9]. There is increasingly a realization that achieving these goals in light of this demographic shift will require more policies focused on child survival and support in low-income countries [10], and these policies must include safe surgery. An analysis of pediatric surgical admissions in Gambia revealed an incidence of surgical problems of 543 per 10,000 children aged 0–14 years with 46% requiring a surgical procedure, and an estimated cumulative risk for all surgical conditions of 85% by age 15 years [11]. Thus, safe and timely surgical care for children is of particular importance in regions with the most limited access to it.

## 2. Methods

For this narrative review, a literature search of English language publications in Pubmed and Google Scholar of terms including ‘pediatric humanitarian surgery’, ‘humanitarian surgery children’, ‘pediatric surgery mission’, ‘humanitarian surgery’, ‘international health surgery’ (with the latter two restricted to the past 10 years) was performed. In addition, a bibliographic review of the most recent major publication of recommendations for surgical care in LMIC, the Disease Control Priorities, 3rd Ed. Volume 1 ‘Essential Surgery’ [6] was conducted. Titles and abstracts were reviewed for thematic content and included in an integrative fashion.

## 3. Challenges

Surgical humanitarian outreach currently is most often delivered in a short-term application, whether by short-term surgical missions, often 1–3 weeks in length, or by international organizations responding to a crisis, with a plan to deliver emergency services for a limited time period. There are exceptions to these short-term engagements, but the majority of humanitarian surgical care is delivered in this manner. The lack of affordable access to general preventive medicine and acute care, and in some settings the reliance on traditional and ineffective therapies, means that pathology is often advanced significantly by the time a patient reaches a qualified surgical provider, and this is true as well for patients reaching humanitarian providers. Comorbidities that will impact a patient’s perioperative course may or may not be diagnosed in advance, and infectious diseases that will impair optimal recovery from surgery, such as HIV, malaria, and tuberculosis, may be prevalent. In children, malnutrition and anemia can be common, and disrupt their perioperative course. Any intervention that takes place will likely occur in a setting of poverty, which limits the ability of patients and families to pay for care and appropriate follow-up. Indeed, each year, 81.3 million people suffer financial catastrophe from necessary surgery [12]. Local support services may be non-existent or under-resourced and ill-equipped to handle complications and contingencies of surgical intervention. Pediatric surgeons are scarce in many parts of Africa, for instance, with a range of one surgeon for a population of 13 million in Malawi to 120 for a population of 80 million in Egypt [13]. A survey of 26 African countries revealed a median waiting time of 40 days for elective procedures and 7 days for emergencies [14]. For foreign providers who travel to perform surgery, there are language and cultural barriers that when not addressed appropriately can inhibit not only the success of a mission but also the likelihood of teams being invited to return in the future. When combined with the political and societal unrest that can be found in developing nations, these factors underscore the complexity of humanitarian surgical investment and the importance of thoughtful interventions and strategies to alleviate the burden of surgical disease in these communities.

## 4. Types of Humanitarian Outreach

Despite these challenges, great strides have been made to provide charitable surgical care for children in under-resourced areas. Activity in humanitarian surgical outreach for children can be broadly divided into four categories. These include humanitarian efforts mobilized and deployed for disaster and crisis relief, temporary short-term surgical platforms including efforts focused on sending teams to treat specific conditions or perform certain operations and self-contained methods of surgical delivery, long-term efforts more explicitly aimed toward specialty facility development, and efforts with a primary focus on training and capacity building (Table 1). There is considerable overlap between these care delivery platforms, and training and capacity building, for instance, frequently occur as part of each platform. Despite this limitation, this categorization has been used elsewhere to separate delivery platforms into those that may be more or less effective in a given context [15]. The next section will elaborate on these categories with an emphasis on applications in pediatric surgery.

### 4.1. Disaster and Crisis Relief

For many, humanitarian surgery brings to mind images of disaster relief and war zone interventions with teams of qualified providers from high-income countries sent to address acute escalations in surgical need. Indeed, disasters may affect up to 200 million children per year [16]. The International Committee of the Red Cross (ICRC) and Médecins Sans Frontiéres (MSF), also known as Doctors Without Borders, are the foremost example of this type of international humanitarian aid organization. The ICRC was founded in 1863 and provides protection to communities impacted by armed conflict as an independent, impartial and neutral humanitarian organization. In 2015, the ICRC supported 476 hospitals in 29 countries where over 132,000 operations were performed [17]. MSF was founded in 1971 to provide humanitarian aid to distressed communities, similar to the ICRC with the added provision of bearing witness to those suffering to provoke political change regardless of the impact on neutrality. MSF currently employs over 36,000 physicians, nurses, engineers, community health workers and administrators. In addition to acute crisis relief, the work of MSF includes food relief, training, education, sanitation improvement, and other health-related activities that include long-term in-country programming. In crisis situations, an important proportion of their services are directed toward children. An analysis of surgical care delivered at MSF facilities in 21 countries showed that 21% of patients were younger than 18 years old and concluded that the “aspiring humanitarian surgeon must consequently be familiar with the management of pediatric patients and be comfortable with performing common pediatric surgical procedures” [18,19]. An example of disaster relief provided not by international non-governmental organizations but by a national government is the US Naval Ship Comfort, which provided surgical care to 237 pediatric patients over 37 days after the January, 2010, earthquake in Haiti [20].

When external governments, international organizations, and non-governmental organizations (NGOs) intercede in disaster and crisis situations, they are often able to provide an acute response that would otherwise be impossible. The coordination of multiple teams of unknown numbers arriving to a devastated setting can cause logistical issues that occasionally cause redundancy and even harm [21]. The establishment of effective policies and protocols that dictate how a coordinated emergency response should unfold has improved this redundancy. The Global Emergency Medical Team developed by the WHO has established minimum standards for international health workers and coordinates leadership structure, training, and quality improvement for over 100 teams ready for swift deployment [22]. The mission of crisis relief is narrowly focused on immediate relief and with appropriate planning, experience, and execution, they are often successful in addressing needs that would otherwise overwhelm local resources that are often already stretched thin. Given the nature of their mission, however, these types of efforts are often limited in the areas of capacity-building, because their focus is specifically not development. Educational opportunities may exist for local providers, but these are commonly spontaneous, volunteer-specific and not organized. Follow-up for individual patients may be difficult and the scope of the mission is necessarily confined to the most immediate, short-term needs. This type of humanitarian intervention has an important role in crisis situations, but other means of sustainable development and external involvement in pediatric surgical care are needed, as well.

### 4.2. Short-Term and Self-Contained Missions for Children

The majority of humanitarian pediatric surgery outreach is provided by temporary surgical platforms in the form of short-term surgical trips or self-contained surgical platforms to deliver surgical care in areas of need [15]. The most common model is a visiting short-term surgical team arriving to an area with a high burden of specific surgical disease, operating for one to two weeks, often bringing the entirety of their own equipment with them [23]. These types of missions may have long-term impact by providing recurring services to the same area over time or may intervene in a more limited scope. Many of these missions treat children—a recent survey of 46 organizations performing more than 220,000 surgical procedures showed that 54% reported performing pediatric operations [24]. Surgeons working with organizations such as Smile Train or Operation Smile, for example, perform thousands of cleft lip and palate repairs, annually. More complex procedures, such as pediatric neurosurgical interventions, have also been provided in a mission-based format with low complication rates and adequate follow-up [25]. Most often, these services are delivered in cooperation with local hospitals or clinics, with patients recruited or screened ahead of time by local providers who may also provide follow-up care. Organizations also commonly seek approval by and partnerships with ministries of health and forge ongoing relationships for recurring missions [26]. ‘Self-contained surgical delivery’ refers to organizations that do not utilize or require local infrastructure or resources to complete their mission and can perform complex care in a high quality, controlled setting [27]. Mobile hospitals, such as those provided by Mercy Ships or the US Navy, carry the entirety of a surgical care infrastructure, including advanced care units and personnel. A variation of this model is shown in the Moore Pediatric Surgery Center in Guatemala City. This dedicated local facility is used by visiting teams to deliver services and has hosted 42 visiting surgical teams who have performed 2260 operations from 2011–2014 [28].

Although short-term missions remain common, nearly 95% of publications on this model lack any significant data collection [29]. While evidence regarding the practice and performance of short-term missions is limited, in general, and for children, in particular, there does exist controversy regarding the efficacy, cost-effectiveness, and sustainability of this model, especially for complex surgical care [15]. The majority of international surgery organizations perform fewer than 500 operations per year [24], a level of care that is at odds with the notion that increased volume is associated with improved outcomes [30]. Outcomes in surgical missions vary by the type of surgical procedure, with simpler procedures showing outcomes similar to high-income settings but more complex procedures, such as cleft palate repair, demonstrating up to 20-fold high complication rates [31]. Cost-effectiveness has been shown in cleft lip and palate repair with an expense as low as $52/DALY averted [32], but data is limited and often does not account for complications and alternative surgical options [15]. Furthermore, while the vast majority of surgical organizations incorporate training and education into their missions [24], this may be counterbalanced by stresses on local infrastructure and distortion of local health markets and systems [33]. A recent modeling study investigating the impact of nine policy changes for surgical access on health benefit, cost, and equity in Uganda found that the ‘mission trip’ platform for surgical care delivery was more expensive and less effective than nearly all other platforms studied [34]. An additional limitation of this model is the lack of harmonization that stems from multiple distinct organizations functioning within the same space without coordination of their efforts, which is a condition common in international aid and addressed by the Paris Declaration on Aid Effectiveness [35]. This disharmony leads to duplication of efforts and resource wasting and remains a significant hindrance to efficiency in short-term missions. Collaborations are in development to improve communication between humanitarian organizations. The Global Paediatric Surgery Network (www.globalpaediatricsurgery.org), for instance, was launched in 2010 with the aim of providing a network between volunteer surgeons for the purpose of enhancing service, education, advocacy, and research collaboration [36].

### 4.3. Specialty Pediatric Surgery Facilities

Pediatric humanitarian surgery has also taken the form of specialty hospitals run and maintained by humanitarian outreach groups which often utilize local providers supplemented by visiting teams or surgeons. In contrast to short-term models, the scope of these efforts is uniformly long-term and often coordinated more closely with local governments, ministries of health, health science education, and supply chains with financing and organizational support provided by a humanitarian agency. There are many examples of internationally funded general hospitals in low-income settings providing surgical care for children. Increasingly, there are specialty hospitals dedicated specifically to the surgical care of pediatric patients. An example of this is CURE International, a non-profit organization which operates pediatric specialty hospitals focused on clubfoot repair, hydrocephalus management, and other common pediatric surgical conditions in 28 low- and middle-income countries. Beit CURE International Hospital in Malawi, which treated 9842 pediatric patients over a ten-year period, is an example of this type of hospital [37].

While the effectiveness of specialty surgery facilities for pediatric-specific conditions has not been reported, the data for cataract-specific facilities has shown that between surgeries performed at a government surgical camp, a medical college and a nongovernmental specialty facility, the nongovernmental facility provided the greatest benefit due to improved quality at a moderate cost [38]. This effectiveness likely comes from the ability of a specialized facility to achieve higher quality from repetition and standardization with economies of scale to control costs. They may also have a horizontal influence on the development of related health and social infrastructure, as these hospitals are embedded within communities that derive benefit from local economic investment and activity. Specialized hospitals may also, however, be fewer in number and have access limitations that prevent widespread adoption as a primary platform for humanitarian surgical care delivery.

### 4.4. Capacity Building and Academic or Society Partnerships

Capacity building should be a key goal for humanitarian outreach in low-resource settings. Equipping local providers to deliver high-quality and affordable care often involves investment in education and training in a new era of Global Surgery, as proposed by the Lancet Commission. While the platforms mentioned above often incorporate training of local providers, there are initiatives in humanitarian surgery in which the primary goal is capacity building and not service delivery. Many North American teaching hospitals have developed relationships with overseas institutions, and the number of programs focused on surgical training in low-income settings continues to expand [39]. Recent broad-based programs have included Human Resources for Health in Rwanda [40], which recruits nearly 100 US faculty members annually to provide medical education across multiple specialties including anesthesia and surgery with a goal of training 500 specialty and subspecialty physicians and 5000 nurses by 2018. Pediatric-specific training programs have been supported by various anesthesia and surgical societies including the World Federation of Societies of Anesthesiologists and the Pan-African Association of Christian Surgeons, which have established fellowships in pediatric anesthesia and surgery in low-income settings with significant financial and training support from partners in high-resource countries. Many non-governmental organizations that provide pediatric surgical services have also incorporated education into their core mission, including Operation Smile, Inc. which has modified its large-scale international team mission model into a model fostering local capacity building and sustainability through educational partnerships at cleft surgery centers where 67% of care is provided locally [41].

Humanitarian outreach in global pediatric surgery education has not been extensively evaluated, but it does provide several key advantages over service-oriented activities. A ranking of collaboration priorities by 96 African surgeons revealed direct clinical care as the lowest and collaborative professional development as the highest of priorities [14]. Empowering local providers over time will allow for sustainable development and encourage local investment, making the need for humanitarian intervention obsolete. Educational missions can be broad in scope depending on the skills of the providers involved and also tend to have a longer focus of engagement as relationships necessary for advanced educational programs require time to develop and produce results. This longer timeframe for generating outcomes is a primary disadvantage of the educational approach, which also may direct resources away from direct patient care in order to support educators and students. The costs of these programs may be substantial, as well—estimates for total training costs for a single specialist physician in a low-income environment range from $75,000 to $150,000 and may comprise 8.5% to 17% of total costs of surgical system scale-up in these settings [42]. Unfortunately, no direct comparison of the costs associated with other platforms in humanitarian outreach and educational initiatives is available.

## 5. Recommendations

Although evidenced-based recommendations are unfortunately not available [29], we make the following recommendations based on available literature and our own experience to optimize program delivery and outcomes. The content is divided into recommendations for establishing new short-term surgical missions and advanced considerations for organizations with well-established programming. This section is intended as a basic framework and comprehensive mission and organizational planning must be context-specific.

In any short-term mission, safety and outcome optimization should be prioritized, and mission participants must have expertise in the conditions they will manage. This is a critical consideration in pediatric surgery, where exposure to index cases encountered in LMICs may be limited after training [43]. For those involved in planning new missions, it is critical to have an understanding of available resources at the destination from the outset [44]. This includes physical equipment, medications, and personnel. It is advisable to have a local contact person that is able to help with these preparations. It must be remembered that access to electricity, internet, and email may be very limited, and this contact may take a considerable amount of time to answer any questions that are posed. Language may also be a critical barrier. It is important to begin this process as soon as the mission is put into place.

Given the complexities of providing care in a foreign environment, teams that have established on-going working relationships are preferable to a new team assembled solely for the purpose of a mission. If the mission involves participants who do not normally work with each other, it is valuable to have a team meeting or conference call prior to the trip. This meeting helps in several ways—it allows connections to be made within the group, but more critically, it allows for an inventory of equipment and medications to be taken. This helps prevent unnecessary duplications or omissions of critical items. In instances where a medical team has partnered with a host facility or institution to provide training experience for visiting team members in the setting of humanitarian service delivery, the nature of the mission should be clearly stated and focused on mutual benefit as outlined in guidelines produced by the Working Group on Ethics Guidelines for Global Health Training [45].

It is critical to include the local health care providers, both from a global health diplomacy standpoint, but also to increase their education as well [44]. There are things to be learned from both sides of the table. In these situations, when a patient has travelled for days to receive care, it can be exceptionally difficult to deny that patient surgical care. Even in the most austere situations, it is important to ensure as much quality as possible, which may require data collection. If data is collected, it should be reported back to the local contact along with any output from that data.

As emphasized above, providing an educational component to local providers through the mission has considerable benefit. Allowing them to ask questions, providing textbooks if possible, and providing clinical teaching can enhance their engagement. Providing educational experiences to the local health care providers can boost local capacity, and may be enhanced by periods of in-country collaboration longer than 1–2 weeks or recurrent missions with the same partners.

In contrast to some small-scale or newly established surgical missions, well-established international humanitarian organizations, such as ICRC or MSF, have the above components fully embedded within their program structure, enabling them to provide effective care in the areas they serve. For organizations focused on service delivery, deficiencies may exist, however, in the areas of training and outcome measurement. While no organization should be expected to excel across the whole range of possible interventions in humanitarian outreach, training and outcome measurement are areas that warrant special focus due to their potential for capacity building, accountability and improvement. In addition to training courses in war surgery and crisis management, for example, an expansion of formalized educational programs specific to pediatric surgery utilizing the extensive experience of ICRC and MSF would be of great benefit to the communities they serve and the international community at large. Similarly, although it may require the addition of research infrastructure or oversight, tracking standardized post-operative outcomes for organizations providing humanitarian surgical outreach could drive quality improvement and enhance transparency. The 24-h post-operative mortality rate (POMR) is one example of a quality metric that is easily measured but infrequently reported and is a valuable tool to assess program performance at a basic level.

## 6. Conclusions

The emerging focus on improving access and quality of surgical care delivered globally by the year 2030 [4] has brought new attention to the status of humanitarian outreach, and especially pediatric surgical outreach. Reflecting international trends in medical aid and outreach, the future of humanitarian surgical outreach for children should include a greater emphasis on transitioning from service delivery to global partnerships and capacity building. Further research into platforms of care provision that provide cost-effective improvement in access and quality for children is needed to inform investment, and this research may support a continued shift to incorporate local development into humanitarian outreach. Gathering the necessary human and material capital required to address the needs of pediatric surgical care will require global partnerships. Given the large discrepancy between the volume of surgeries that are needed in low-resource settings and the local capacity that exists to meet that need, it is critical that those invested in the care of children in LMICs continue to advocate for more international cooperation and effort in pediatric surgery and ensure that the gains in child health and quality of life achieved in high-income settings are realized everywhere.

## Figures and Tables

**Table 1:** A summary of surgical outreach types with advantages, disadvantages, educational focus and examples of each. Of note, examples cited often engage in multiple types of outreach.

Type of Outreach	Advantages	Disadvantages	Educational Focus	Examples
***Disaster and crisis relief***	Narrow focus, rapid deployment, specially equipped	Limited capacity building, often short-term scope	Education and training of local providers is frequent, but not usually structured	International Committee of the Red Cross (ICRC); Médecins Sans Frontiéres (MSF); US Naval Ship Comfort
***Short-term/self-contained missions***	High total volume and participation, specialty care available.	Outcome variability, often high expense, stress to local infrastructure	Education and training often included in the mandate, and may be structured	Operation Smile; Mercy Ships; Moore Pediatric Surgery Center Guatemala; Surgical Eye Expeditions International; Children’s Surgery International
***Specialty hospitals***	Cost effective, local investment and development.	Access limitations, large upfront and ongoing costs	Education for surgeons and anesthesia providers is usually available and structured	CURE International, Bethany Kids
***Capacity building and academic partnerships***	Human resource development, long-term scope	Limited short-term impact, high operational costs	Education and training are central to the mandate and mission	Smile Train, Human Resources for Health in Rwanda, Hôpital Universitaire de Mirebalais, Haiti (Partners in Health)
